# Assessing the Relative Contributions of Active Ankle and Knee Assistance to the Walking Mechanics of Transfemoral Amputees Using a Powered Prosthesis

**DOI:** 10.1371/journal.pone.0147661

**Published:** 2016-01-25

**Authors:** Kimberly A. Ingraham, Nicholas P. Fey, Ann M. Simon, Levi J. Hargrove

**Affiliations:** 1 Center for Bionic Medicine, Rehabilitation Institute of Chicago, Chicago, Illinois, United States of America; 2 Department of Physical Medicine and Rehabilitation, Northwestern University, Chicago, Illinois, United States of America; Peking University, CHINA

## Abstract

Powered knee-ankle prostheses are capable of providing net-positive mechanical energy to amputees. Yet, there are limitless ways to deliver this energy throughout the gait cycle. It remains largely unknown how different combinations of active knee and ankle assistance affect the walking mechanics of transfemoral amputees. This study assessed the relative contributions of stance phase knee swing initiation, increasing ankle stiffness and powered plantarflexion as three unilateral transfemoral amputees walked overground at their self-selected walking speed. Five combinations of knee and ankle conditions were evaluated regarding the kinematics and kinetics of the amputated and intact legs using repeated measures analyses of variance. We found eliminating active knee swing initiation or powered plantarflexion was linked to increased compensations of the ipsilateral hip joint during the subsequent swing phase. The elimination of knee swing initiation or powered plantarflexion also led to reduced braking ground reaction forces of the amputated *and* intact legs, and influenced both sagittal and frontal plane loading of the intact knee joint. Gradually increasing prosthetic ankle stiffness influenced the shape of the prosthetic ankle plantarflexion moment, more closely mirroring the intact ankle moment. Increasing ankle stiffness also corresponded to increased prosthetic ankle power generation (despite a similar maximum stiffness value across conditions) and increased braking ground reaction forces of the amputated leg. These findings further our understanding of how to deliver assistance with powered knee-ankle prostheses and the compensations that occur when specific aspects of assistance are added/removed.

## Introduction

The number of individuals with lower-limb amputations living in the U.S. is projected to grow dramatically [1], and common clinical options for these individuals include mechanically-passive prosthetic knees and feet. These devices provide swing phase resistance through friction, pneumatic and/or hydraulic mechanisms and normally lock during the stance phase of walking. Some of these systems are microprocessor-controlled, which can regulate their passive mechanics using more complex control architectures. Yet, the biomechanical benefits of these more advanced systems in the gait of transfemoral amputees have been mixed (e.g., [2, 3, 4]). There are also mechanically-active commercial [5, 6] and research prototype systems [7], which can better replicate the force-generating capabilities of muscles. Muscles spanning the knee and ankle have important roles for regulating the acceleration of the body during gait [8, 9]. Thus, when an individual receives a transfemoral amputation, the loss or physical modification of these muscles presents a significant and challenging task of replacing these biological characteristics with non-biological prosthetic systems.

A significant challenge is that there are an infinite number of ways to generate mechanical energy at the knee and ankle joints of a prosthesis during walking. For example, a variety of physical hardware options such as actuator type and strength, etc. (e.g., [10]) are complemented by various active control options such as joint impedance based architectures (e.g., [7, 11, 12]), artificial reflex and positive force feedback models (e.g., [13]), virtual constraint [14] and quasi-stiffness [15]. All of these approaches have been proposed and tested in human subjects, with encouraging results. They are usually evaluated in the context of the resulting biomechanics, and frequently compare knee and ankle kinematics and kinetics to those of able-bodied non-amputees. There have also been studies aimed at characterizing angle-dependent stiffness of the biological knee (e.g., [16]) and ankle (e.g., [17, 18]), and methods to replicate the passive properties of these joints with active or quasi-active systems, such as the use of series elastic actuators in knee prostheses [19, 20] and pneumatic cylinders to produce non-linear stiffness profiles in a prosthetic ankle [21]. Assessing how specific aspects of either passive or active control strategies contribute to a given movement is a challenge and has infrequently been attempted with empirical methods. Rather, theoretical, simulation-based approaches have been used [22–24]. Thus, in large part, it remains unknown how various ways of providing assistance at the prosthetic knee *and* ankle joints affect the walking mechanics of amputees. This is especially true when examining outcomes in *transfemoral amputees* and during *overground* walking. Interesting prior studies have sought to emulate autonomous prosthetic systems with externally-powered devices and treadmill-based test beds to answer some of these questions regarding the plantarflexive assistance occurring during the mid- to late stance phase of walking (e.g., [25]). While providing promising insights, these studies have been typically performed on able-bodied subjects who simulate an amputated pathology by wearing an orthotic boot that constrains ankle movement and attaching a device to the distal end of the boot.

Little is known about how combinations of knee *and* ankle assistance influence the walking mechanics of *transfemoral amputees*. With the rapid advances in powered prosthetic hardware and the encouraging prior studies that demonstrate the potential benefits of these devices [26, 27], there is a need to better understand how the control of these devices influences the biomechanics of the user. The purpose of this study was to investigate how providing powered ankle plantarflexion and active flexion of the knee (i.e., knee swing initiation) in terminal stance and increasing ankle stiffness throughout stance contribute to the walking mechanics of unilateral transfemoral amputees. These findings could motivate the refinement of prosthesis physical designs and controllers, incorporating various combinations of active and passive knee and ankle assistance. In this study, we compared kinematics and kinetics of both the amputated and intact legs from a group of transfemoral amputees as they wore an active knee and ankle prosthesis that provided various combinations of knee and ankle device assistance. We hypothesized that providing powered plantarflexion and knee swing initiation would increase the joint power generation of the prosthetic ankle and knee as well as decrease the power generation of the ipsilateral hip joint in the second half of stance. We also hypothesized that providing powered plantarflexion and knee swing initiation would offload the intact leg, and specifically would reduce the frontal-plane knee moment and ground reaction force. Finally, we hypothesized that increasing ankle stiffness throughout stance would influence the moment profile of the ankle during stance and have little influence on joint power.

## Methods

### Participants and design of experiments

**Fig 1:**
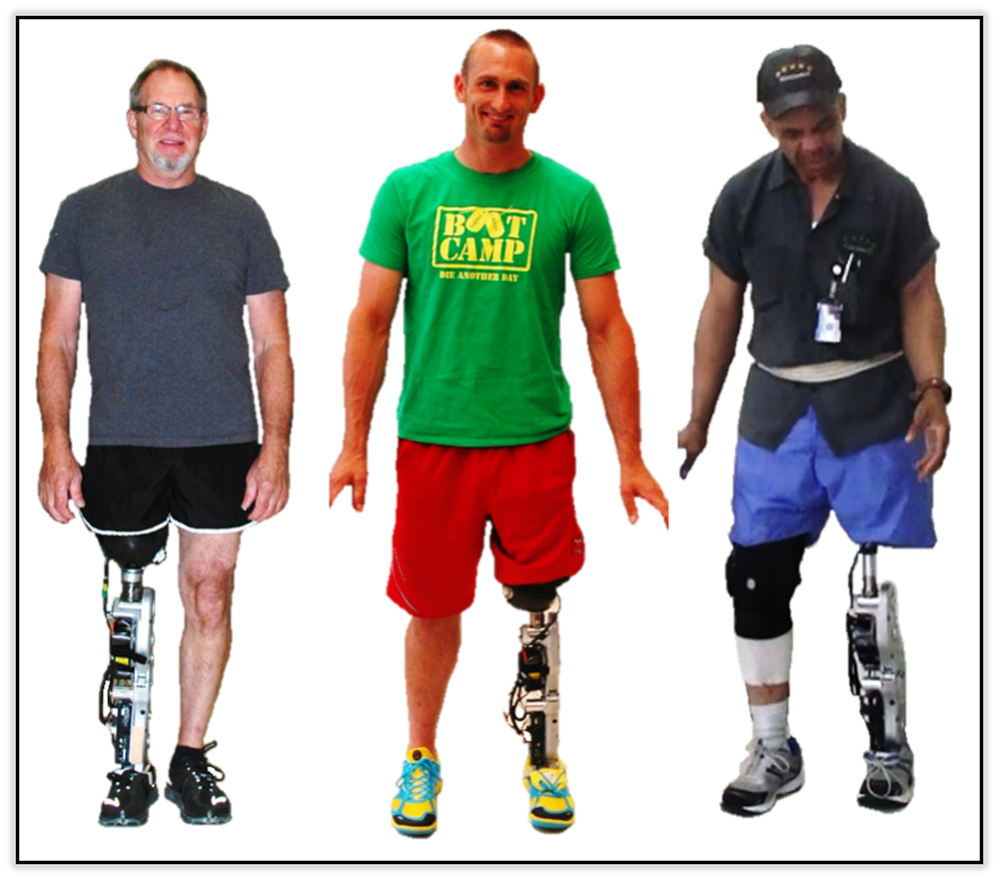
Transfemoral amputee subjects wearing the powered knee and ankle prosthesis.

Three male subjects with unilateral amputations at or above the knee (87.5 ± 5.1 kg, weight; 1.8 ± 0.1 m, height; 51 ± 19 years, age; 34 ± 15 years, time post-amputation; Fig 1) participated in the study after providing written informed consent to an approved Northwestern University IRB protocol and written informed consent (as outlined in PLOS consent form) to publish these case details. All subjects were community ambulators with Medicare functional classification levels K3 or K4.

**Fig 2:**
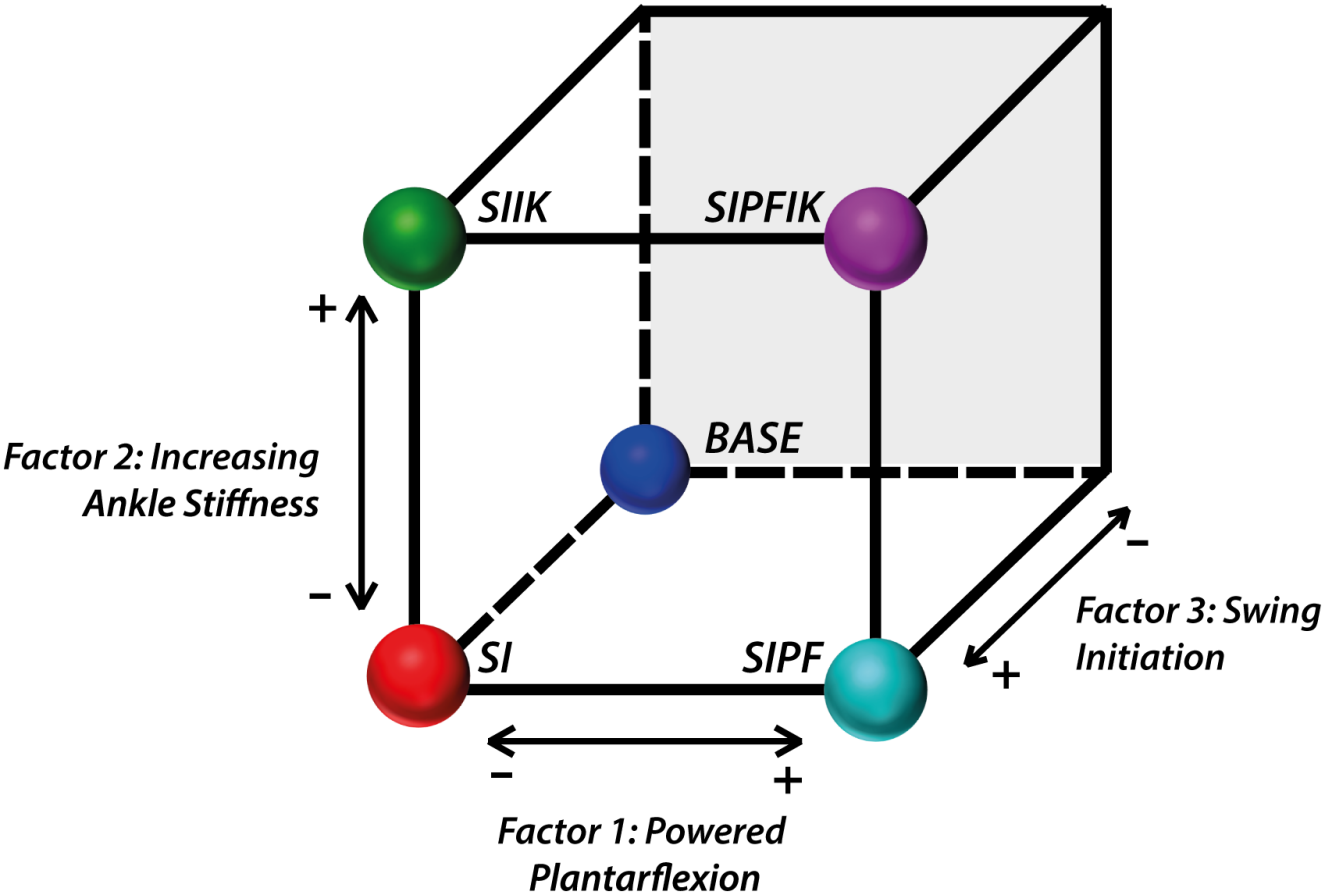
Design of experiments. A partial factorial design was used to evaluate the contributions of three factors: powered plantarflexion, increasing ankle stiffness and knee swing initiation.

A design of experiments was formulated to assess the contributions of three factors at two levels: with or without powered plantarflexion, increasing ankle stiffness and knee swing initiation (Fig 2). Walking without knee swing initiation was challenging for subjects; therefore only one condition was tested at this level. The design resulted in five conditions tested for each subject, featuring different combinations of knee and ankle assistance during the stance phase. The baseline condition (BASE) did not provide any energy to the user during stance phase (i.e., no knee swing initiation, powered plantarflexion, or increasing ankle stiffness). The four remaining conditions featured either knee swing initiation alone (SI), or in combination with increasing ankle stiffness (SIIK), powered plantarflexion (SIPF), or both (SIPFIK). The order of conditions was randomized across subjects.

For each condition, subjects were allowed time to accommodate to the device settings. Subjects walked overground at their self-selected pace along an 8-m walkway with three embedded force plates (Advanced Mechanical Technology, Inc.). Repeated trials were conducted until five clean force plate contacts were captured for both amputated and intact legs. Ground reaction force (GRF) and kinematic data (sampled at 1 kHz and 100 Hz, respectively) were collected using an eight-camera motion capture system (Motion Analysis Corp.). For each subject, reflective markers were placed on the posterior sacrum and bilaterally on the posterior superior iliac spine (PSIS), anterior superior iliac spine (ASIS), greater trochanter, lateral and medial femoral epicondyle, lateral and medial malleolus, posterior heel, dorsal foot, and fifth metatarsal head. Clusters of three markers affixed to thermoplastic shells were wrapped bilaterally to the thigh and shank.

### Device and control strategies

**Fig 3:**
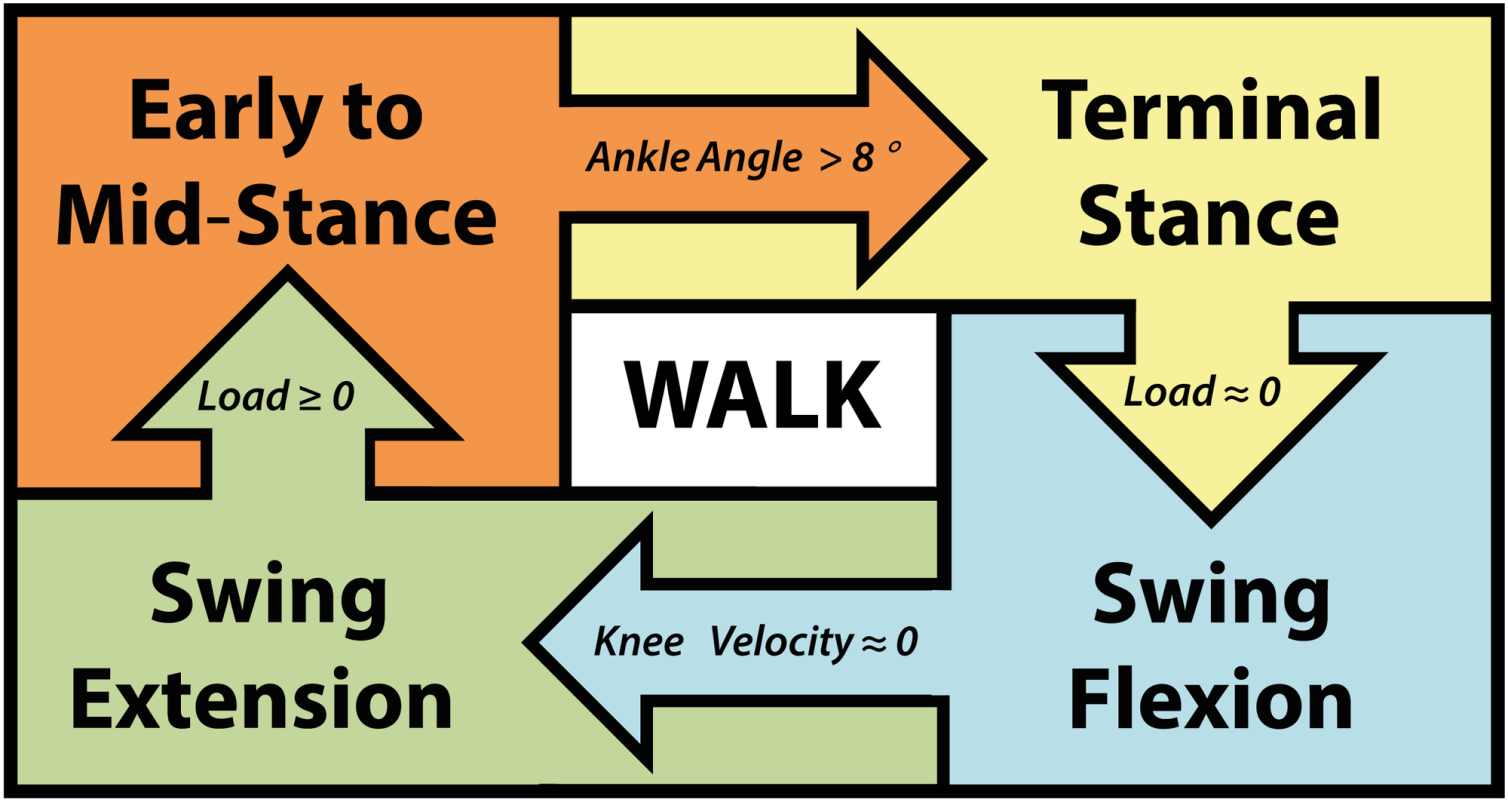
Diagram of the finite state machine used to control walking.

Subjects wore a powered knee and ankle prosthesis featuring on-board mechanical sensors, including sensors to measure knee and ankle position and velocity, as well as axial load applied through the shank [12]. Sensor data were streamed at 500 Hz and collected using custom software. The prosthesis also included a custom carbon fiber foot, a foot shell, and a shoe. The device was controlled using an impedance-based model, which generated torque, *τ*, at the knee and ankle joints as a function of three impedance parameters: joint stiffness, *k*, equilibrium angle, *θ*_e_, and damping coefficient, *b*, according to Eq 1.

$$\tau_{i}=-k_{i}\left(\theta_{i}-\theta_{ei}\right)-b\dot{\theta },$$ (1)

where *i* corresponded to the knee or ankle joint, *θ* was the joint angle and $\dot{\theta }$ was the joint angular velocity. Walking was controlled using a finite state machine comprised of four states: early to mid-stance, terminal stance, swing flexion and swing extension (Fig 3). Transitions between states were controlled by thresholds on specific mechanical signals. The majority of impedance parameters in each state were set to constant values, but knee and ankle stiffness and equilibrium angle parameters were modified across conditions in early to mid-stance and terminal stance to provide knee swing initiation, powered plantarflexion, and increasing ankle stiffness.

**Fig 4:**
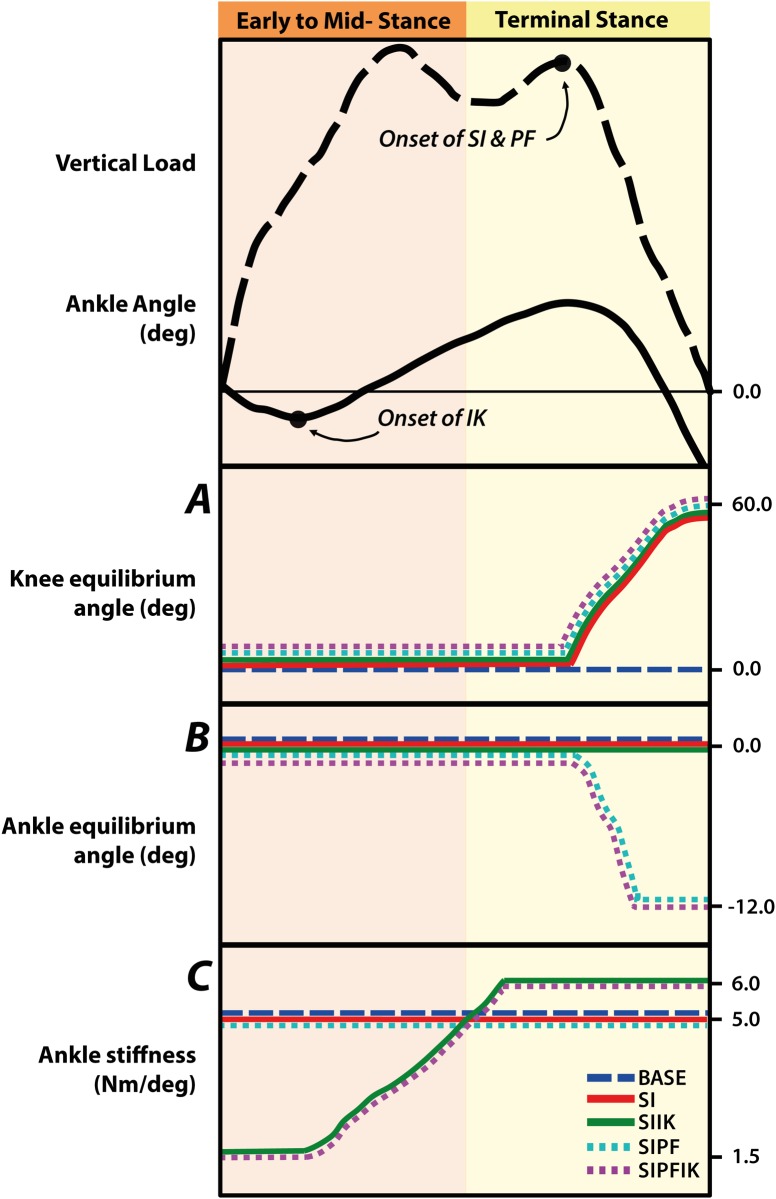
Impedance parameters modified across conditions. A) Knee equilibrium angle, B) ankle equilibrium angle, and C) ankle stiffness were modified in early to mid-stance and late stance. Increasing ankle stiffness was initiated in early to mid-stance when the ankle angle (solid black line) began to dorsiflex. Swing initiation and powered plantarflexion were initiated in late stance when the vertical load (dashed black line) began to decrease.

To provide knee swing initiation, the prosthesis knee equilibrium angle was increased from 0° to 60–75° flexion (ranged across subjects) during terminal stance as a linear function of decreasing prosthesis vertical load [11]; the stiffness of the knee joint was also decreased in this manner [11]. In the condition without knee swing initiation, prosthesis knee equilibrium angle and knee stiffness remained constant throughout stance phase (Fig 4A). To achieve powered plantarflexion, the prosthesis ankle equilibrium angle was increased from 0° to 12° plantarflexion during terminal stance as a linear function of decreasing prosthesis vertical load [11]. In conditions without powered plantarflexion, the ankle equilibrium remained neutral (0°) throughout stance phase (Fig 4B). In conditions with increasing ankle stiffness, the prosthesis ankle stiffness increased as a linear function of ankle dorsiflexion angle (Fig 4C), as previously tested [11] and originally based on characterizations of biological ankle stiffness in able-bodied subjects [18]. Ankle stiffness was capped at a value of 6 Nm/deg, as to not saturate the torque capabilities of the device. In conditions without increasing ankle stiffness, the ankle stiffness remained constant throughout stance phase at 5 Nm/deg, the value of the increasing ankle stiffness equation evaluated at the transition between early to mid-stance and terminal stance states (Figs 3 and 4C). Prior to the experiment, all subjects had >15 hours prior experience walking with the prosthesis, controlled according to the SIPFIK condition. This condition was previously tuned for each subject according to a protocol involving input from the patient, engineers and clinicians to ensure adequate swing clearance and patient comfort at a self-selected speed [12].

### Analyses

Spatiotemporal, kinematic and kinetic data were analyzed using Visual3D (C-Motion, Inc.) and Matlab (Mathworks, Inc.). Kinematics were segmented from heel contact to heel contact; kinetic data were segmented from heel contact to toe-off (stance phase) and toe-off to heel contact (swing phase), and normalized to body mass (kg). All data were low-pass filtered and averaged across strides. Average peak values were calculated for joint kinematics. Average positive and negative values were calculated for GRF and joint kinetics during stance, and also calculated for joint kinetics during swing. To provide objective comparisons of these metrics across all conditions, across-subject statistics were performed (SPSS 22, IBM, Corp.). For each quantity, two analyses of variance with repeated measures (ANOVAs) were evaluated (α = 0.05). One two-way ANOVA tested the effects of powered plantarflexion and increasing ankle stiffness. The second tested the effect of swing initiation. Post-hoc tests using a Bonferroni correction factor were used to identify differences between conditions.

## Results

### Spatiotemporal parameters

Increasing ankle stiffness had a significant effect on stance time of the intact leg (p = 0.02), with a trend toward reduced stance time. There were no differences in step length, step time, swing time, overall cycle time, walking speed, stride length, or stride width for either amputated or intact legs (Table 1).

**Table 1:** Stride spatiotemporal parameters.

		Condition				
Parameter		BASE	SI	SIIK	SIPF	SIPFIK
Step Length (m)	*Amputated*	0.65 ± 0.10	0.71 ± 0.09	0.71 ± 0.16	0.71 ± 0.10	0.70 ± 0.03
	*Intact*	0.55 ± 0.05	0.58 ± 0.04	0.59 ± 0.05	0.58 ± 0.05	0.56 ± 0.04
Step Time (s)	*Amputated*	0.75 ± 0.04	0.69 ± 0.07	0.67 ± 0.05	0.69 ± 0.06	0.68 ± 0.07
	*Intact*	0.65 ± 0.06	0.63 ± 0.06	0.62 ± 0.11	0.62 ± 0.08	0.62 ± 0.08
Stance Time (s)	*Amputated*	0.88 ± 0.05	0.83 ± 0.12	0.80 ± 0.11	0.84 ± 0.09	0.84 ± 0.08
	*Intact* §	0.97 ± 0.08	0.89 ± 0.11	0.87 ± 0.12	0.88 ± 0.10	0.87 ± 0.09
Swing Time (s)	*Amputated*	0.52 ± 0.04	0.49 ± 0.04	0.49 ± 0.06	0.48 ± 0.05	0.46 ± 0.03
	*Intact*	0.53 ± 0.19	0.43 ± 0.06	0.44 ± 0.06	0.44 ± 0.05	0.44 ± 0.04
Cycle Time (s)		1.39 ± 0.09	1.32 ± 0.17	1.30 ± 0.18	1.31 ± 0.14	1.31 ± 0.11
Speed (m/s)		0.87 ± 0.10	0.99 ± 0.17	1.04 ± 0.25	1.00 ± 0.18	1.01 ± 0.15
Stride Length (m)		1.20 ± 0.06	1.29 ± 0.09	1.33 ± 0.15	1.30 ± 0.11	1.31 ± 0.08
Stride Width (m)		0.21 ± 0.04	0.20 ± 0.02	0.21 ± 0.03	0.21 ± 0.03	0.22 ± 0.03

^§^ Significant effect of increasing ankle stiffness

### Ground reaction forces

**Fig 5:**
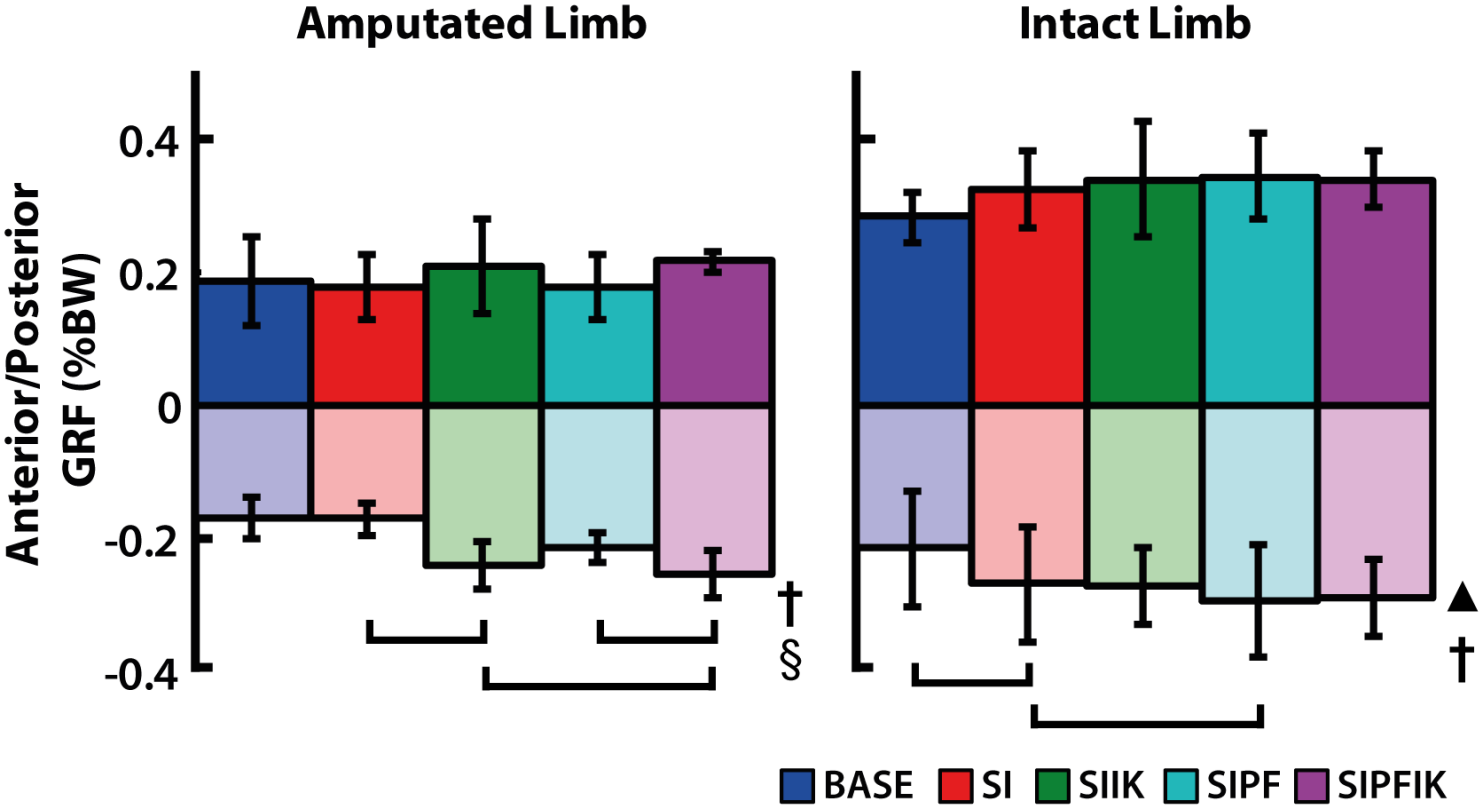
Anterior/posterior (A/P) GRF for amputated and intact limbs. Average positive and negative A/P GRF values are shown. Main significant effects of swing initiation (▲), powered plantarflexion (**†**), and increasing ankle stiffness (§) are denoted. Significant pairwise t-test results are indicated with brackets.

Average posterior GRF (i.e., braking) of the *amputated* leg was affected by increasing ankle stiffness (p = 0.023) and powered plantarflexion (p = 0.006; Fig 5). Conditions with increasing ankle stiffness had increased braking with and without powered plantarflexion (SIPFIK vs. SIPF, p = 0.042; SIIK vs. SI, p = 0.021). In the absence of increasing ankle stiffness, powered plantarflexion resulted in increased braking (SIPF vs. SI, p = 0.004). Average braking of the *intact* leg was also affected by powered plantarflexion (p = 0.001; Fig 5). In the absence of increasing ankle stiffness, powered plantarflexion resulted in increased braking (SIPF vs. SI, p = 0.040). Knee swing initiation also resulted in increased braking of the intact leg (SI vs. BASE, p = 0.002). No differences were found in average propulsive GRFs of the amputated and intact legs.

### Joint kinematics

**Fig 6:**
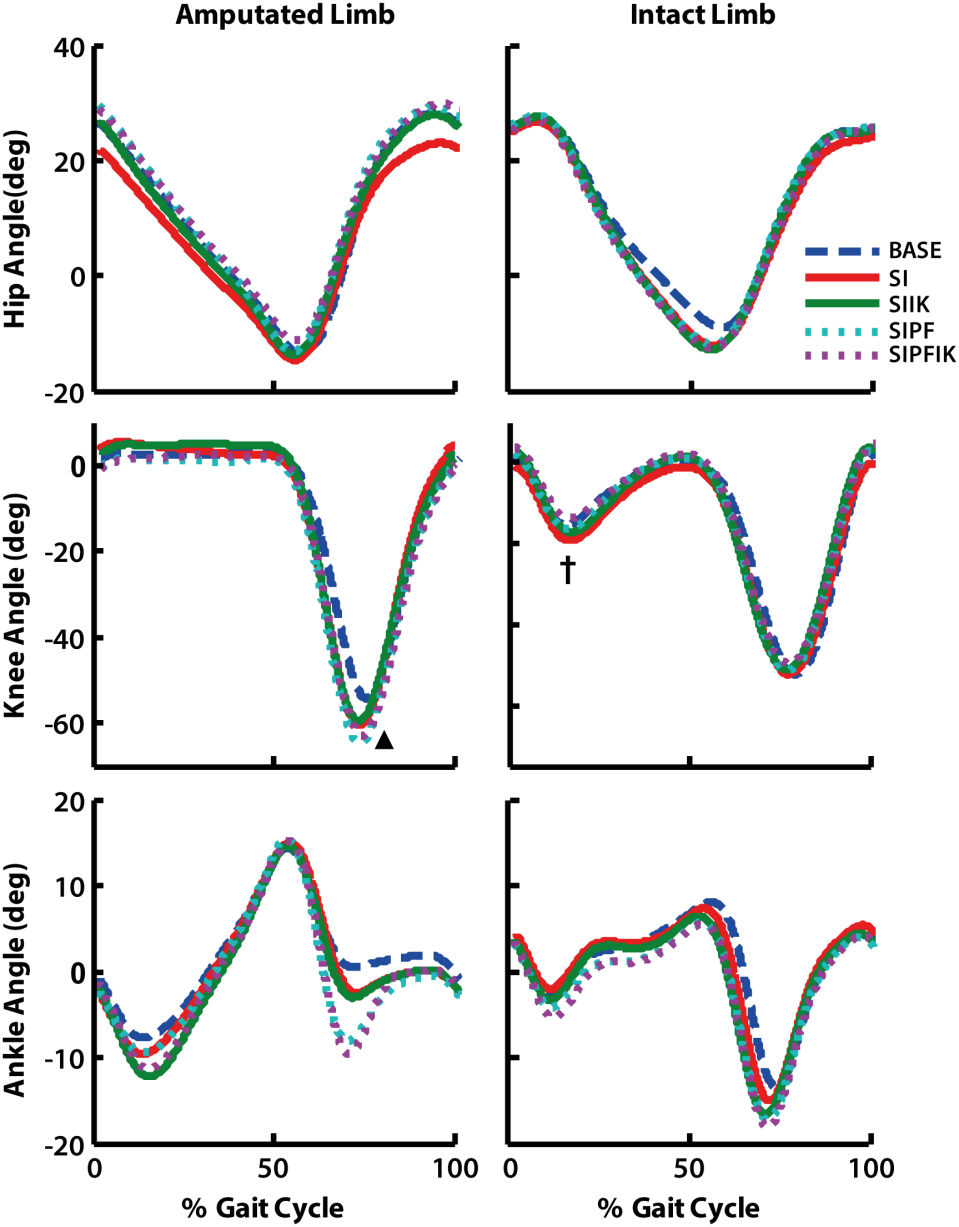
Kinematics for hip, knee, and ankle joints of the amputated and intact limbs. Group average results shown. Main significant effects of swing initiation (▲) and powered plantarflexion (**†**) are denoted.

Powered plantarflexion had a significant effect on the peak stance phase knee flexion angle of the intact leg (p = 0.002), with a trend toward less knee flexion with powered plantarflexion. Knee swing initiation increased peak knee flexion of the amputated leg in swing (SI vs. BASE, p = 0.008; Fig 6).

### Joint kinetics of the amputated leg

**Fig 7:**
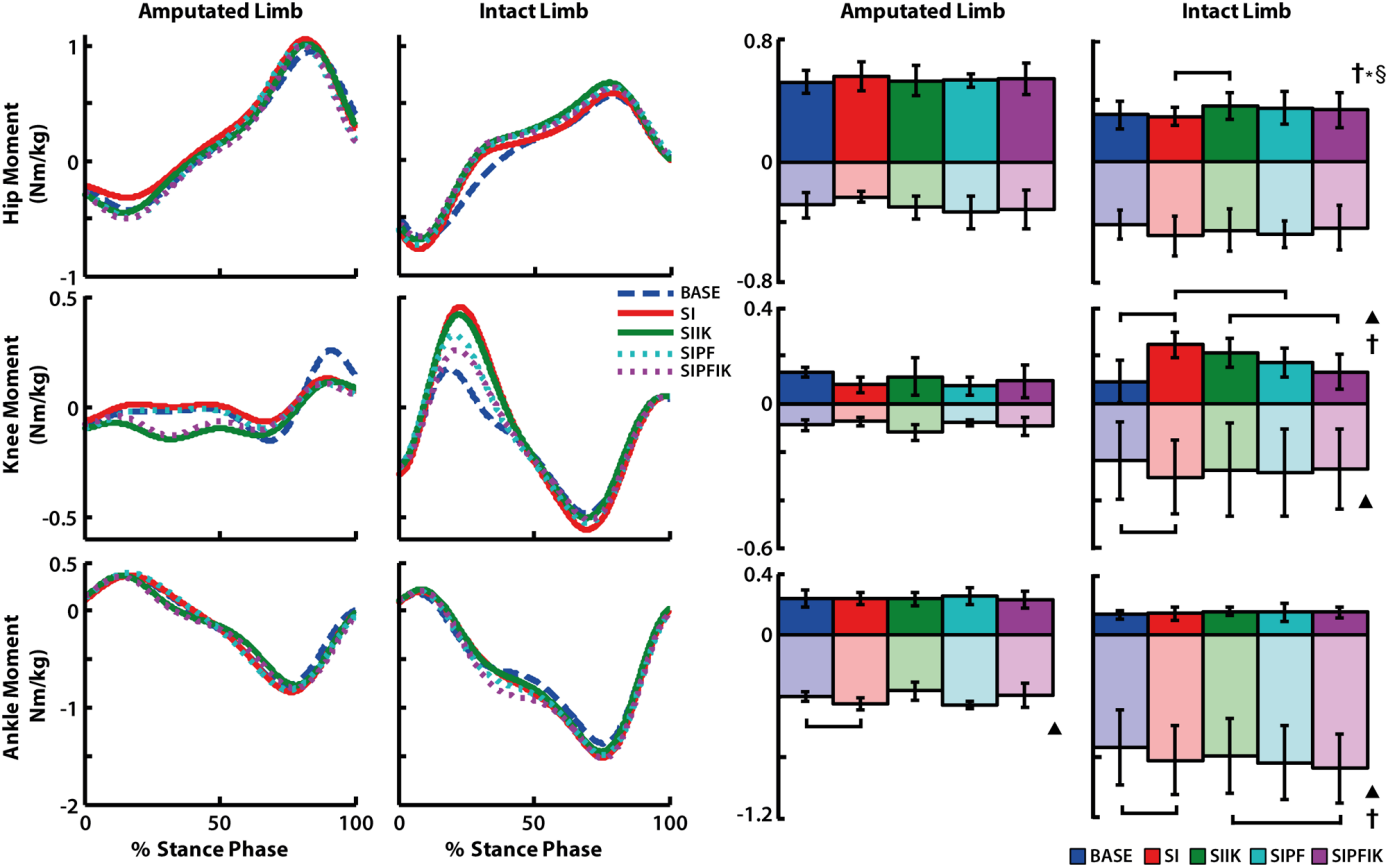
Stance phase joint moments for hip, knee, and ankle of the amputated and intact limbs. Group average traces and average positive and negative values of joint moments are shown. Significant main effects of swing initiation (▲), powered plantarflexion (**†**), and significant interaction effects of powered plantar flexion and increasing stiffness (†*§) are denoted. Significant pairwise t-test results are indicated with brackets.

**Fig 8:**
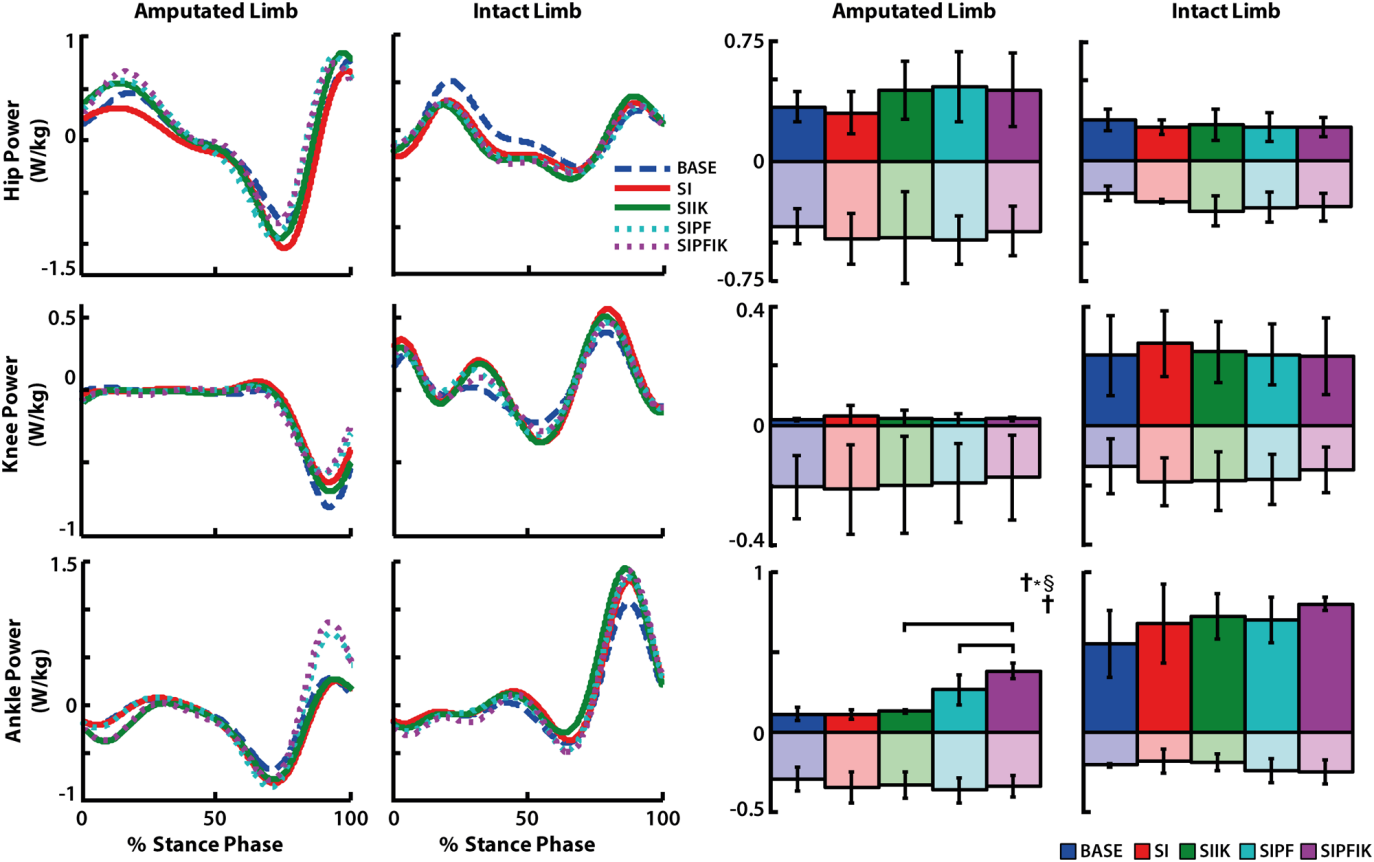
Stance phase joint power for hip, knee, and ankle of the amputated and intact limbs. Group average traces and average positive and negative values of joint power are shown. Significant main effects of powered plantarflexion (**†**) and significant interaction effects of powered plantar flexion and increasing stiffness (†*§) are denoted. Significant pairwise t-test results are indicated with brackets.

During stance of the amputated leg, knee swing initiation significantly increased average plantarflexion moment (SI vs. BASE, p = 0.009; Fig 7). Average positive ankle power was significantly affected by powered plantarflexion (p = 0.021), and the interaction between powered plantarflexion and increasing ankle stiffness (p = 0.033; Fig 8). Providing both powered plantarflexion and increasing ankle stiffness resulted in higher positive ankle power than either powered plantarflexion or increasing stiffness alone (SIPFIK vs. SIPF, p = 0.050; SIPFIK vs. SIIK, p = 0.007).

**Fig 9:**
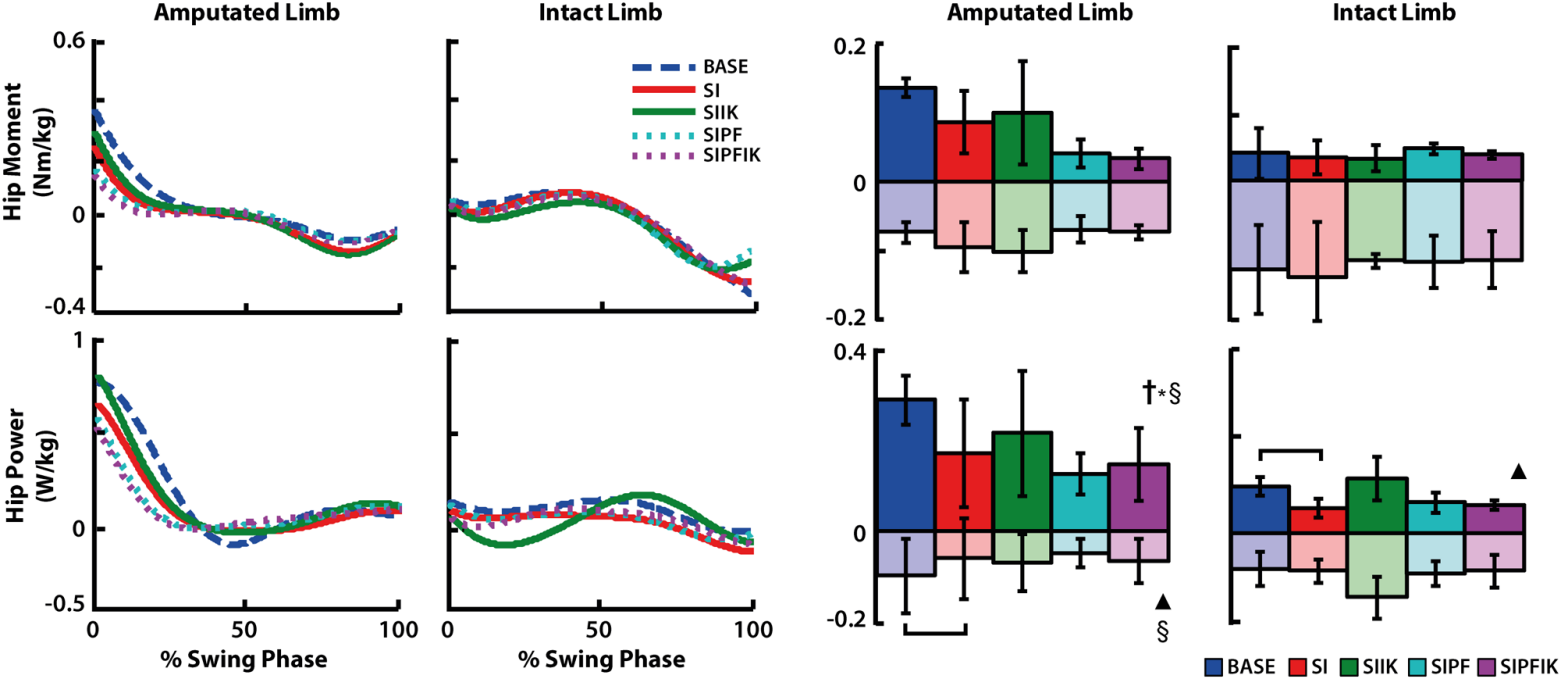
Swing phase hip moment and power of the amputated and intact limbs. Group average traces and average positive and negative values of hip moment and power are shown. Significant main effects of swing initiation (▲), powered plantarflexion (**†**), and increasing ankle stiffness (§), and significant interaction effects of powered plantar flexion and increasing stiffness (†*§) are denoted. Significant pairwise t-test results are indicated with brackets.

During swing of the amputated leg, increasing ankle stiffness had a significant main effect on average negative hip power (p = 0.050), and powered plantarflexion and increasing ankle stiffness had a significant interaction effect on average positive hip power (p = 0.018; Fig 9). Knee swing initiation resulted in increased negative hip power (SI vs. BASE, p = 0.028; Fig 9).

### Joint kinetics of the intact leg

**Fig 10:**
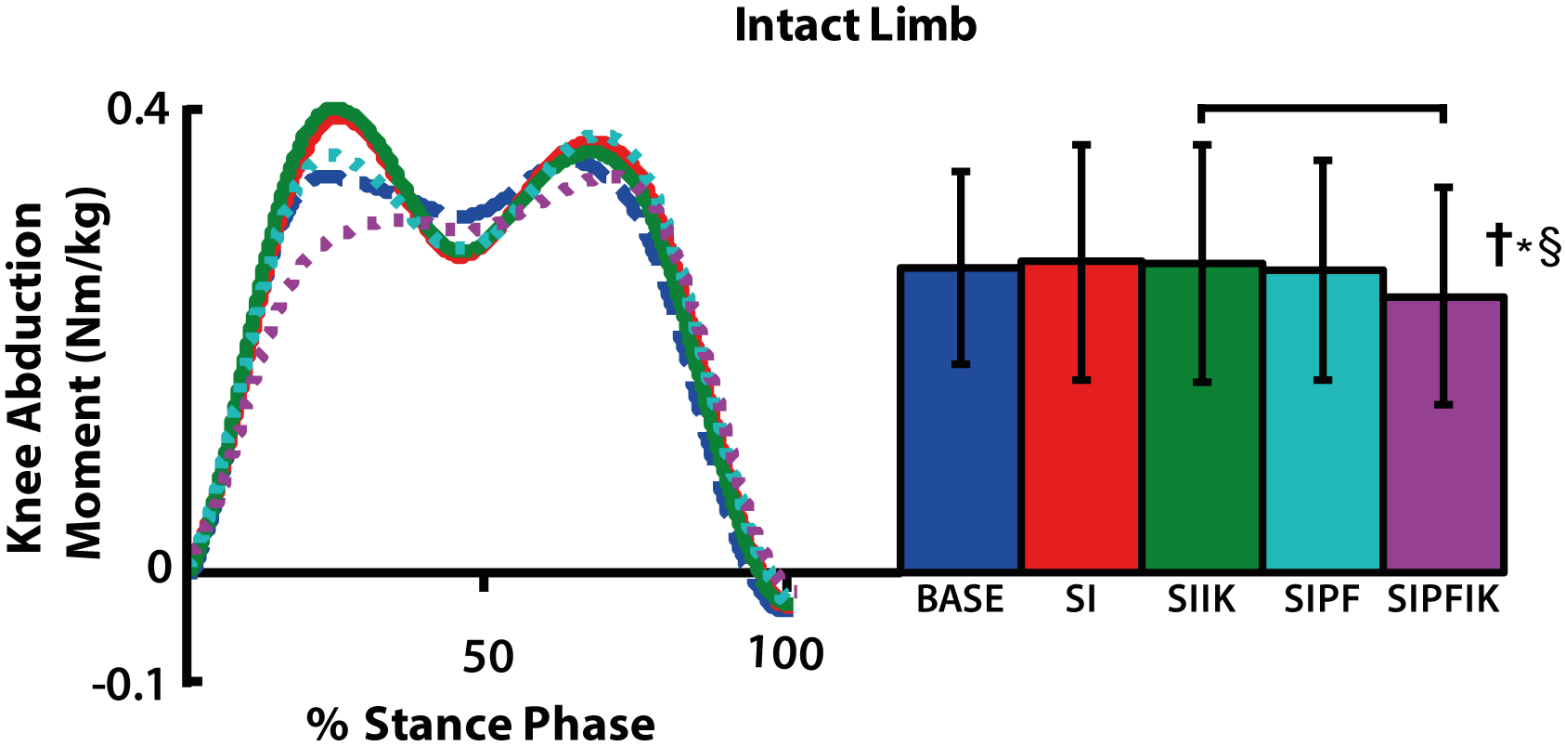
Stance phase intact knee abduction moment. Group average traces and average positive values of knee abduction moment are shown. Significant interaction effects of powered plantar flexion and increasing stiffness (†*§) are denoted. Significant pairwise t-test results are indicated with brackets.

During stance of the intact leg, knee swing initiation resulted in increased average knee extension moment (p = 0.023), knee flexion moment (p = 0.043), and ankle plantarflexion moment (p = 0.022; Fig 7). Powered plantarflexion also had a significant main effect on average knee extension moment (p = 0.006) and ankle plantarflexion moment (p = 0.023). Conditions with powered plantarflexion had decreased knee extension moment with and without increasing ankle stiffness (SIPFIK vs. SIIK, p = 0.011; SIPF vs. SI, p = 0.005), and increased plantarflexion moment in the presence of increasing ankle stiffness (SIPFIK vs. SIIK, p = 0.031). Powered plantarflexion and increasing ankle stiffness had a significant interaction effect on average hip flexion moment (p = 0.037; Fig 7) and knee abduction moment (p = 0.006; Fig 10). In the absence of powered plantar flexion, increasing ankle stiffness resulted in increased hip flexion moment (SIIK vs. SI, p = 0.039). In the presence of increasing ankle stiffness, powered plantar flexion resulted in decreased average knee abduction moment (SIPFIK vs. SIIK, p = 0.032).

During swing of the intact leg, knee swing initiation resulted in decreased positive hip power (SI vs. BASE, p = 0.049; Fig 9).

## Discussion

This study investigated how providing powered ankle plantarflexion, increasing ankle stiffness and knee swing initiation influences the walking mechanics of unilateral transfemoral amputees. Several trends emerged that were both supportive and unsupportive of our hypotheses.

### Eliminating knee swing initiation influences swing-phase hip mechanics of the amputated leg

Predictably, eliminating swing initiation resulted in delayed and less overall knee flexion of the prosthesis during early swing (Fig 6). We hypothesized that providing knee swing initiation would also reduce positive power of the ipsilateral hip during terminal stance. These changes were unsupported. However, altering knee swing initiation did influence the mechanics of the hip during the subsequent swing phase (Fig 9). Without knee swing initiation, trends toward more positive hip power in early swing and more negative hip power in late swing were observed. Muscle groups that flex the hip and knee act to deliver energy to the leg in terminal stance and early swing [8, 28–30]. Thus, these data support the idea that in the absence of active knee flexion in terminal stance, there was increased demand on the hip to accelerate the leg forward. But, this compensation was found to occur in early swing, rather than late stance. These data also support the idea that the roles of muscles and/or actuators spanning the hip and knee to deliver energy to the leg should be examined over an extended duration spanning both terminal stance and swing, as opposed to a more isolated view of these functions occurring within a very specific region of the gait cycle. Even less predictably, in the absence of knee swing initiation, there was increased demand on the hip to absorb greater energy in terminal swing (Fig 9), likely to absorb greater leg energy (e.g., [30]). Similar responses have been observed in studies that added inertia to the leg of able-bodied non-amputees, which found increases in hip flexion and extension moments during swing phase [31]. Studies have not shown that eliminating active knee flexion in terminal stance influences *both* flexion and extension of the hip of transfemoral amputees during swing.

### Eliminating powered plantarflexion also influences swing-phase hip mechanics of the amputated leg

Modifications to powered plantarflexion were also shown to have an effect on the positive power of the ipsilateral hip (Fig 9). These changes also occurred during the subsequent swing phase, and conditions providing powered plantarflexion appeared to reduce the positive power of the hip in early swing (Fig 9). Studies have shown that the net effect of the uniarticular soleus in able-bodied non-amputees or plantarflexion moment generated by passive prostheses is to absorb energy from the leg over the second half of stance [8, 22, 24]. But, these contributors also have the potential to deliver energy to the leg at the very end of stance [22], which would be consistent with these changes observed in early swing.

Thus, consistent with our first hypothesis, providing both knee swing initiation and powered plantarflexion in terminal stance influences power generation of the ipsilateral hip. But, inconsistent with our hypothesis, these changes do not occur until the subsequent swing phase. Similar results have been shown when providing increasing levels of powered plantarflexion to non-amputees with an externally-powered prosthetic ankle emulator. As those individuals walked on treadmill, increasing levels of powered plantarflexion also decreased positive hip power of the ipsilateral leg in late stance and early swing [25].

### Eliminating knee swing initiation is compensated for with reduced intact leg braking

Consistent with our second hypothesis, providing powered plantarflexion and knee swing initiation influenced loading of the intact leg. Although, some loads (GRFs, moments and powers), were decreased and others increased. We found in the absence of knee swing initiation, these transfemoral amputees compensated by reducing the braking (i.e., posterior) ground reaction force of the intact leg (Fig 5). This finding appears to implicate increasing activity of the intact leg hamstring muscles in early stance or decreased early activity as well as prolonged activity of the quadriceps in stance. Decreased extension and flexion moments of the knee in the intact leg during the first half of stance were also shown (Fig 7). The quadriceps contribute to braking, and the hamstrings can contribute to propulsion if active in early stance [30, 32]. Either of these mechanisms would explain the compensations observed in this study when knee swing initiation was eliminated. Previously, a tendency of transtibial amputees to reduce the braking ground reaction force of the *amputated* leg in the absence of active ankle assistance has been shown [33]. In this study, eliminating knee swing initiation was shown to contribute to reduced braking of the *intact* leg.

### Providing powered plantarflexion reduces sagittal-plane moment of intact knee, and reduced braking of both intact and amputated legs is a mechanism to compensate for a lack of powered plantarflexion

Similar to the changes noted above with eliminating knee swing initiation, reduced braking of the *intact* leg also occurred with a lack of powered plantarflexion in the presence of knee swing initiation (Fig 5). Though, unlike the changes observed when knee swing initiation was eliminated, the elimination of powered plantarflexion did not correspond with a decreased intact knee extension moment. Rather, consistent with our second hypothesis, *providing* powered plantarflexion decreased (i.e., offloaded) the knee extension moment in early stance, irrespective of ankle stiffness (Fig 7). The ankle plantarflexors and plantarflexion moment have been shown to contribute to forward propulsion and vertical support of the body [8, 28, 34]. Thus, an explanation is that in the powered plantarflexion conditions, the prosthetic ankle is providing more propulsion and support, and thus the role of the intact leg (specifically the knee) to deliver these functions when the prosthesis is trailing and intact leg leading is decreased. This explanation is also supported by the predictable result that powered plantarflexion increased positive power of the prosthetic ankle during terminal stance (Fig 8), as well as the more interesting finding that these large increases in ankle power were shown to result in a significant trend toward less overall knee flexion of the intact leg knee during stance (Fig 6). Similar theories have been presented regarding the reduction of step-to-step transitional work of the leading intact leg when powered plantarflexion is provided by the trailing prosthetic ankle of transtibial amputees [35], which would also support these data.

The elimination of powered plantarflexion also decreased the braking ground reaction force of the *amputated* leg in conditions with constant ankle stiffness (Fig 5). Decreased amputated leg braking ground reaction forces have been identified as an important mechanism to provide increased net propulsive ground reaction force impulses as walking speed increases in transtibial amputees walking with passive prosthetic feet [33]. Thus, an interpretation of this finding is that the removal of powered plantarflexion in transfemoral amputees results in the prosthetic ankle contributing less to forward propulsion of the body [22, 34].

This study shows that reducing the braking ground reaction force of the intact *and* amputated legs appear to be important mechanisms for increasing net propulsion in the absence of powered plantarflexion in transfemoral amputees. In addition, providing powered plantarflexion can reduce sagittal-plane loading of the knee joint in the intact leg.

### Stance phase ankle stiffness influences shape of stance phase ankle moment and power generation

In support of our third hypothesis, gradually increasing ankle stiffness influenced the stance phase ankle moment. But, these characteristics mostly influenced the shape of the ankle moment profile during mid-stance, rather than the average magnitude (Fig 7). A lower initial stiffness and subsequent gradually increasing stiffness in this region of the gait cycle resulted in a decreased dorsiflexion moment and sharper increase in plantarflexion moment, as well as an overall shape that was less linear during this region. This shape of the prosthetic ankle moment more closely resembled the shape of the intact ankle moment profile (Fig 7), which is significant since the relationship between ankle stiffness and dorsiflexion angle (i.e., rate of increasing ankle stiffness) was generated by a study that delivered angular perturbations to the ankles of able-bodied non-amputees during overground walking [18]. In contrast to our third hypothesis, in the presence of powered plantarflexion, gradually increasing ankle stiffness resulted in higher positive ankle power during stance (Fig 8). This finding was unexpected since the maximum value of ankle stiffness was similar in both increasing and constant ankle stiffness conditions. This finding suggests that an altered ankle control strategy preceding powered plantarflexion can influence how powered plantarflexion is performed. In this study, powered plantarflexion was provided by modifying the equilibrium position of the ankle as a linear function of the decreasing axial force (initial value and rate of decrease) registered in the device during terminal stance [11]. Thus, powered plantarflexion is adaptable to the initial force conditions, how the device is unloaded, and the configuration of the body in terminal stance. Within this construct, increasing ankle stiffness was shown to influence both the shape of the ankle moment, with a sharper rate of ankle plantarflexion moment, *and* power generation in late stance. These results further highlight how modifications of device control in a particular region of the gait cycle can influence walking biomechanics within that region, as well as how modifications can contribute to changes that are propagated later in the gait cycle.

### Reduced ankle stiffness in early stance increases braking ground reaction forces of amputated leg

With and without powered plantarflexion, gradually increasing prosthetic ankle stiffness was shown to result in increased braking GRFs of the amputated leg (Fig 5). A potential mechanism for these increases in braking was that the foot achieved “foot-flat” faster due to lower initial ankle stiffness. Indeed, a trend toward that result was observed with increased plantarflexion angle of the prosthesis in early stance (Fig 6). A prior study which altered the stiffness of passive prosthetic feet in transtibial amputee walking showed that when foot stiffness was decreased, braking of the amputated leg increased [36]. Then, using modeling approaches, a follow-up study found when stiffness was decreased, the increased braking GRFs were due to the foot contributing more to braking of the leg and less to forward deceleration of the trunk [24]. Our findings are supportive of a similar mechanism occurring when ankle stiffness of a powered prosthesis is decreased in early stance of transfemoral amputees, irrespective of powered plantarflexion.

In the absence of powered plantarflexion, gradually increasing ankle stiffness also resulted in the hip of the intact leg producing a moment profile that was biased more flexor during the last two thirds of stance (Fig 7), relative to the constant stiffness condition. These changes largely occurred when the amputated leg was leading and the intact leg was trailing, when prosthetic ankle stiffness was lower. Thus, a decreased ankle stiffness of the prosthesis in early stance may have allowed muscles spanning the hip of the intact leg to contribute more to swing initiation of the intact leg (e.g., [30]), if the ankle joint of the leading prosthetic leg contributed less to forward deceleration of the trunk, as described above.

### Offloading the intact knee joint in frontal plane via coupled prosthetic knee and ankle assistance

We found that the frontal plane loading of the knee can be decreased via coupled assistance at the ankle and knee (Fig 10). Specifically, a combination of all three factors tested: powered plantarflexion, increasing ankle stiffness and knee swing initiation reduced the knee abduction moment of the intact leg during stance. A decrease in the first peak of the knee moment would correspond in time with providing powered plantarflexion, and prior studies investigating transtibial amputee gait have shown that providing powered plantarflexion with a prosthetic ankle can decrease this quantity [37, 38]. Our study shows this also occurs in transfemoral amputees. In addition, our study suggests that the combined effect of all three factors may reduce the second peak of the knee moment profile (Fig 10).

The magnitude of the knee abduction moment has been linked to disease severity of patients who suffer from osteoarthritis of the medial compartment of the knee joint [39], as well as the progression of knee osteoarthritis in symptomatic patients [40]. Given that transfemoral amputees have an abnormally high prevalence of developing knee osteoarthritis in their intact knee as a long term consequence of limb loss, reducing loading via the coupled assistance of the knee and ankle is important. Interestingly, these decreases in magnitude of mechanical loads at the intact knee were accompanied by a trend toward decreased loading duration (p = 0.10). Descriptively, the longest stance time on the intact leg was observed with a lack of both knee swing initiation and powered plantarflexion (Table 1). Thus, combined knee and ankle assistance in terminal stance may also decrease the impulsive (duration dependent) loading properties of knee joint in the intact leg of transfemoral amputees.

### Limitations and future considerations

There are a number of limitations of the current study that should be considered. First, there are basic limitations such as the use of an experimental prototype and a small sample size, due to our preference for testing experienced users of the device. In addition, a finite number of conditions could be tested due to time and exertion considerations of these subjects. We would have preferred a full-factorial investigation of the three factors, for a total of eight conditions (e.g., Fig 2). But, we limited the total number of conditions to five to avoid patient fatigue. Also, we would have liked to introduce a range of different ankle stiffness profiles (e.g., [36]), or levels and timing of power delivery (e.g., [41]). As constructed, the experiment was an “all or nothing” investigation. A sweep of different control parameters (e.g., [25]) would also be beneficial. Lastly, although no significant differences in walking speed were found across conditions, speed was not tightly constrained (Table 1). Thus, some counterintuitive results not discussed could be due to a confounding influence of a loosely-controlled walking speed. For example, results such as knee swing initiation influencing ankle plantarflexion moment of the intact leg and powered plantarflexion influencing plantarflexion moment of the intact leg (Fig 7) are difficult to interpret if not for a potential influence of subtle changes in walking speed across conditions.

## Conclusions

Although this study can be considered a preliminary evaluation due to a relatively small sample size, convincing and unique trends were found that revealed compensations that occur bilaterally across multiple joints when specific aspects of active assistance are added or removed. These findings further our understanding of how to deliver assistance to transfemoral amputees walking with powered knee and ankle prostheses. Providing knee swing initiation, increasing ankle stiffness and powered plantarflexion with active prostheses *all* influence the walking mechanics of transfemoral amputees. Namely, eliminating active knee swing initiation or powered plantarflexion of the prosthesis led to increased compensations of the ipsilateral hip joint during the subsequent swing phase of the amputated leg. Eliminating knee swing initiation or powered plantarflexion was also linked to decreased braking ground reaction forces of the amputated *and* intact legs, and influencing both sagittal and frontal plane loading of the knee of the intact leg. With a gradually increasing prosthetic ankle stiffness, the shape of the plantarflexion moment was influenced, more closely mirroring the intact leg ankle moment’s shape; positive power generation of the prosthetic ankle increased, despite a similar maximum stiffness value across conditions; and amputated leg braking ground reaction forces increased, possibly contributing to increased/decreased braking of the leg/trunk.

### Implications

This investigation was framed to answer basic questions pertaining to prosthetic knee and ankle assistance during transfemoral amputee walking. Yet, this investigation also has applied implications. Depending on the specific needs or walking abnormalities of a particular transfemoral amputee patient or sub-group of patients, certain combinations of knee and ankle control could be most beneficial. For example, in patients with increased mechanical loading or secondary joint disease in their intact leg, such as osteoarthritis, all three control factors investigated could be beneficial. Providing powered plantarflexion, increasing ankle stiffness and knee swing initiation produced the smallest frontal plane joint moment of the intact leg knee joint, a quantity that has been associated with osteoarthritis severity and progression. In addition, transfemoral amputee patients frequently exhibit increased metabolic energy expenditure during walking. Providing knee swing initiation and powered plantarflexion could benefit these patients, since not providing these functions were shown to increase joint kinetics of other remaining lower-limb joints (e.g., the ipsilateral hip). While many studies have been focused on reducing the metabolic cost of amputee walking by providing assistance at the ankle joint exclusively, this investigation reveals that the synchronization of ankle and knee power in late stance and monitoring how these changes influence kinetics of proximal joints and joints of the intact leg may be especially important to resolve the increased metabolic cost of amputee walking. Finally, based on the biomechanical outcomes evaluated in this study, when fitting elderly patients or other patients at risk of exhibiting gait instability or sustaining fall-related injuries with active knee-ankle prostheses, control strategies that deliver both active knee and ankle assistance may prove to be detrimental. Powered plantarflexion and knee swing initiation were shown to increase braking ground reaction forces relative to conditions that did not provide these functions, which would likely influence stability in the sagittal plane. Thus, hybrid solutions which selectively deliver active knee or ankle assistance could be beneficial. These implications are subjective, and we also understand that individual patient needs could be coupled and be presented simultaneously. Thus, as mechanically-active devices become more commonplace, studies which comprehensively identify how specific aspects of active knee and ankle control influence the biomechanics of locomotion are likely to become increasingly important.
